# Development of an intervention theory for a mobile mammography unit research project for breast cancer screening

**DOI:** 10.17269/s41997-025-01118-1

**Published:** 2026-04-29

**Authors:** Séverine Beuriot, Timothée Delescluse, Cyrille Delpierre, Guy Launoy, Elodie Guillaume

**Affiliations:** 1 U 1086 INSERM ANTICIPE, Université de Caen, CHU Caen Normandie - Av de la Côte de Nacre, Caen, France; 2 Société Française de Santé Publique, Promotion Santé Normandie, Paris, France; 3 UMR 1295 EQUITY, Toulouse, France

**Keywords:** Interventional research in population health, Mobile health units, Social determinants of health, Behavior change, Breast cancer screening, Theorical framework, Recherche interventionnelle en santé des populations, Unité mobile, Déterminants sociaux de la santé, Changement de comportement, Dépistage du cancer du sein, Cadre théorique

## Abstract

**Setting:**

In France, participation rates in breast cancer screening have been decreasing, along with socio-territorial inequalities. To enhance women’s access to breast cancer screening, an interventional research project was implemented to assess the effectiveness, efficiency, and optimal modalities of a mobile mammography unit for women living remotely in Normandy.

**Intervention:**

The randomized cluster trial was directed towards rural women. Before deploying the mobile unit, an intervention theory was developed. The principles of action to reduce inequalities, the definition and standardization of interventions, and the link to behavior change theories to establish causal mechanisms were integrated into a single model. We aimed to produce new knowledge on these mechanisms and develop intervention evaluations.

**Outcomes:**

Using the key function/implementation/context model, the key functions of the expected intervention were listed, linked to the behavior change technique and theoretical domain framework, and finally to the capability, opportunity, motivation–behavior model components. The mechanisms and levers that can improve access to breast cancer screening are also described. This approach also allowed the standardization of stakeholders’ implications and their corresponding actions. Concrete interventions implemented included scheduling appointments at the mobile unit and implementing a suite of measures to improve the information provided to women regarding breast cancer screening.

**Implications:**

This theoretical framework should be compared with interventions carried out during the deployment of mobile units. Various elements interact dynamically with and alter originally planned interventions. This experiential feedback may inform decisions on the transferability of mobile mammography units to other contexts.

**Trial registration:**

This study was registered at ClinicalTrials.gov (registration date: December 21, 2021; registration number: NCT05164874).

## Résumé

**Contexte:**

En France, le dépistage du cancer du sein fait face à une diminution du taux de participation et à des inégalités socio-territoriales. Afin d’améliorer l’accès des femmes au dépistage, un projet de recherche interventionnelle a été conduit pour évaluer l’efficacité, l’efficience et les conditions optimales d’utilisation d’une unité mobile de mammographie combiné à de l’information ciblée. Le protocole de l’étude, un essai randomisé en clusters a ciblé des zones rurales où les femmes résident à plus de 15 minutes d’un centre de radiologie agréé.

**Intervention:**

En amont du déploiement de l’unité mobile, une théorie de l’intervention a été développée. L’objectif était d’intégrer dans un modèle, les principes d’actions pour réduire les inégalités, la définition et la standardisation des interventions tout en les reliant à des théories de psychologie du changement de comportement afin d’établir des mécanismes causaux, et d’anticiper les moyens d’évaluation.

**Résultats:**

Grâce au modèle Fonction clés/Implémentation/Contexte, les fonctions clés de l’intervention ont été listées et reliées au Com-B Model. Une typologie d’acteurs de terrain et d’actions ont pu être définies. Les interventions concrètes qui ont été implémentées sont la proposition d’un rendez-vous à l’unité mobile et une déclinaison d’actions pour améliorer l’information des femmes sur le dépistage du cancer du sein.

**Implications:**

Ce cadre théorique doit maintenant être confronté aux interventions réellement réalisées durant la tournée de l’unité mobile, certains éléments, qu’il faudra définir, étant venues interagir et modifier les interventions initialement prévues. Ce retour expérientiel permettra d’éclairer la décision sur la transférabilité d’unités mobiles de mammographie.

## Introduction

Like most developed countries, breast cancer remains the leading cancer among women in France, with 60,000 new cases estimated in 2023. It is also the leading cause of cancer-related deaths, with 12,000 deaths (INCA, 2023). Since 2004, breast cancer screening has been conducted nationwide (Organized Breast Cancer Screening). Biennially, average-risk women aged between 50 and 74 years are invited to undergo a clinical breast examination and screening mammography at an approved radiology center. A second reading is conducted in the absence of any detected anomalies. If the results are negative, a new invitation is sent 24 months later. In the presence of a positive finding and the subsequent confirmation of cancer through auxiliary diagnostics, women are integrated into the healthcare management system. The screening participation rate has decreased since 2012. During 2022–2023, the uptake was 46.5%, as reported by Santé Publique France (Rogel et al., 2023), which is below the 70% rate recommended by the European Commission to decrease breast cancer mortality rates.

The factors behind the non-use of breast cancer screening are well known and refer to what is called the social determinants of health (Marmot et al., 2008). These factors include sociodemographic and environmental factors, such as rurality, relationships with the healthcare system, and psychological factors. These factors are socially stratified, and several studies have identified social and territorial inequalities in breast screening participation along a social gradient. Thus, the more socially disadvantaged a woman is and lives in a disadvantaged environment, and/or the further she is from an approved radiology center, the less likely she is to participate in breast cancer screening (Ouédraogo et al., 2015; Rollet et al., 2021).

These unjust and avoidable inequalities are a major public health concern. To reduce these inequalities, initiatives have been developed to enhance the accessibility of breast cancer screening using mobile mammography units (Greenwald et al., 2017; Vang et al., 2018). The concept involves equipping a vehicle with mammography equipment as a radiology center and going out to women who reside far from an approved center. This initiative has been in operation in France since the 1990s. A retrospective assessment of a mobile unit in the Orne Department of Normandy highlighted its efficacy in increasing screening participation and diminishing health inequalities (Guillaume et al., 2017). These units are also found in Europe and America; however, no evaluation of them has been conducted to date (Greenwald et al., 2017; Vang et al., 2018).

The Mammobile project, which was initiated in 2018, is a pioneering interventional study on population health. Its objectives were to assess the effectiveness of a mobile mammography unit on breast cancer screening participation, its efficiency, and its ability to reduce socioterritorial inequalities. This study was designed to define the optimal conditions for utilizing such devices. As part of a cluster randomized controlled trial, the mobile unit was operated from March 2022 to October 2023 in Normandy. The expected evidence-based results may substantially contribute to global reflection and provide policymakers with critical insights into the nationwide implementation of mobile units.

In anticipation of a complex evaluation and to provide new knowledge on the mechanisms to be transferred, an intervention theory was developed for this project (Hawe et al., 2009). This article presents the methodology used to construct the intervention theory. It describes the stakeholder typology and interventions implemented, the evaluation systems conceived to analyze mechanisms, and levers mobilized by women to participate in breast cancer screening.

## Methods

### Objectives

Our objective was to develop a theoretical model to describe how an intervention may enhance the accessibility and utilization of breast cancer screening in our study context. This model requires the consideration of the fundamental principles that act on the social determinants of health. As issues related to information and communication quickly emerged as major issues, the harmonization of stakeholder engagement and informational actions became pivotal. Additionally, we sought to delineate causal mechanism pathways to understand how our interventions worked and to evaluate both the main outcomes and the effectiveness of the supposed mechanisms. Such theoretical models are particularly useful for assessing the effects of complex health interventions and providing relevant insights into their transferability.

### Multidisciplinary workshops

The construction of the intervention theory and the resulting evaluation occurred in a multiphase process facilitated by five multidisciplinary collaborative workshops from September 2020 to March 2022. The inaugural workshop defined the objectives associated with intervention theory. The second was devoted to developing a methodology for constructing an intervention theory. The third focused on information tools, particularly the decision aid tool devised for mobile projects. The fourth workshop was concerned with the creation of questionnaires to assess informed choices in relation to the decision-aid tool, women’s satisfaction with screening in the mobile unit, and the levers mobilized by women to participate. The final workshop provided a synthesis of the four preceding workshops.

These workshops involved numerous collaborators. Regarding participants of the intervention theory construction workshop, in addition to social epidemiology researchers of the two research units implied, one of the researchers was an epidemiologist specialized in shared medical decision-making, one was a health psychologist, specialized in the links between health literacy and shared decision-making, and one was a sociologist studying public health policies and their regional variations. The group also included a project manager from the Normandy Health Promotion Association, which supports public health policies, particularly health promotion. A social work institute also participated in the process development (without participating in the workshop) to list the relevant stakeholders to involve in our interventions in order to better inform women about organized breast cancer screening and the mobile mammography unit. This workshop enabled us to validate and enrich the methodology for constructing the intervention theory. The coordinator ensured the participation of the partners and facilitated the drafting of reports.

### Intervention theory development

The “key functions/implementation/context” model (FIC model) was used to finely describe interventions and enhance their evaluation and transferability. This model allowed the identification of the core components, the “key functions” of an intervention, which represent its transferable dimensions. These are distinguished from the implementation, which refers to the elements of the intervention’s execution (tools, mechanisms, procedures, and resources used). The context refers to the institutional, political, social, or economic environment in which the action takes place, encompassing all external factors that influence implementation. This structured framework makes it possible to implement an intervention across varying contexts. On the other hand, this model helps to prevent social inequalities in health from worsening and even reduces them (Fianu et al., 2017; Villeval et al., 2019).

**Fig. 1:**
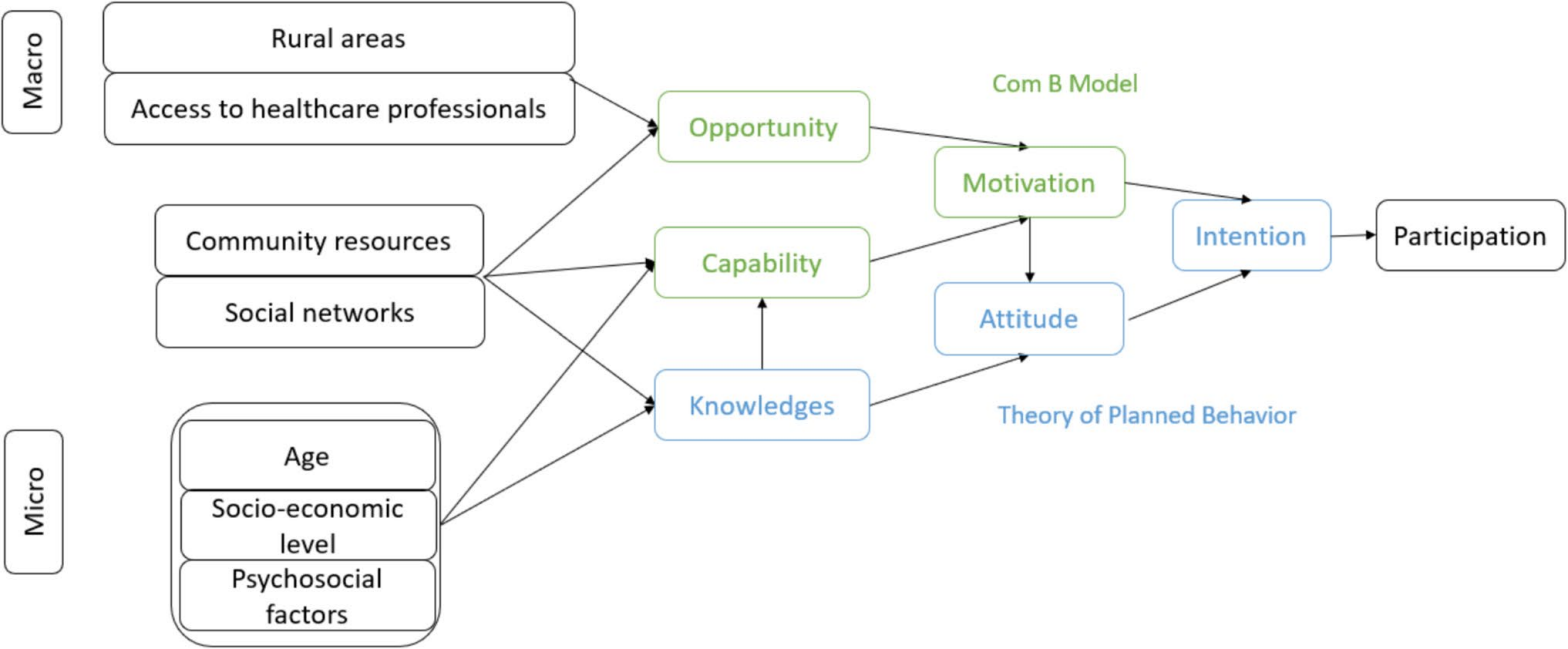
Causal model linking the determinants of non-participation in breast cancer screening, the constructs of the Capability, Opportunity, Motivation-Behavior (COM-B) Model, and the theory of planned behavior to the behavior: participation

A causal model was established. It considered the determinants of non-participation in breast cancer screening as its departure point. An analysis of the literature allowed the identification of behavior change theories that should be incorporated into the model. The COM-B model and the theory of planned behavior were combined to make the link with knowledge, attitude, and intention, as shown in Fig. 1. These determinants were linked to the psychosocial dimensions of the models. The results are presented as a causal diagram describing the mechanisms that could enable women to participate in breast cancer screening through the proposed interventions and mobilizable levers.

Subsequently, the key functions were described. To reduce inequalities and target the social determinants of health, interventions proposed to women integrated the Commission on Social Determinants of Health principles of action recommendations: multi-level interventions, cross-sectoral interventions, context-dependent interventions, interventions that enable women’s empowerment, and implementation according to proportionate universalism (Marmot et al., 2008). Concurrently, a reflection was conducted to define the stakeholder typology and standardize their information actions. The key functions were organized into three tables according to the targets of their actions: actions to strengthen individuals, actions to strengthen the community, and actions to strengthen the conditions of access to screening. The context of each function was also described. A series of concrete actions (implementation) was then declined. Individual reinforcement actions concern women’s information, screening planning, and monitoring. An example of a key function is *multiplying the opportunities for information delivery*. Community reinforcement actions concern stakeholder mobilization and training. An example of a key function is *mobilization of prevention stakeholders*. Reinforcement actions for access to organized breast cancer screening concern organizational and policy levels, with a key function of *facilitating access to services*, corresponding to the mobile unit proposal.

The next step was based on Michie’s behavior change wheel (Michie et al., 2011), developed to guide the design of effective behavior change interventions. The wheel’s core is the COM-B Model. It states that behavior results from the interaction between capacity (C), opportunity (O), and motivation (M). The middle layer comprises nine types of interventions that can influence behavior, and the outer layer comprises higher-level levers that support interventions.

The key functions listed above have been linked to the classification of behavior change techniques (BCTs) (Michie et al., 2015), selected to target the theoretical domain framework (Cane et al., 2012), as well as one or more capability, opportunity, motivation-behavior (COM-B) model dimensions of capability, opportunity, and motivation (Michie et al., 2011). The interventions deployed were designed in accordance with the recommendations for concurrently targeting multiple TDFs, thereby engaging all three components of the COM-B model to promote behavioral change (Tables 1, 2, 3).

**Table 1:** Individual reinforcement actions

Key functions	Context	Implementation	BCT = > TDF = > COM-B model
Taking into account the heterogeneity of the initial level of women’s information on breast cancer screening	-Ill-informed women -Rural areas -Heterogeneity of literacy level	Adaptation/creation of information tools with: - Consideration of the principle of literacy (pictograms, simple sentences) - Principle of numeracy - Ethical principle: information on risks (over-diagnosis, over-treatment)	4: Shaping knowledge = > Knowledge = > Capability 5: Natural consequences = > Knowledge = > Capability 9: Comparison of outcomes = > Beliefs = > Motivation
Providing information on a new screening modality	Mobile unit = new screening modality	Information on the mobile unit has been added as a complementary modality, with a number of points to bear in mind: women retain the right to choose their screening location, mobile unit screening complies with the same specifications as screening in a radiology center, and sufficient time is devoted to each woman	4: Shaping knowledge = > Knowledge = > Capability 5: Natural consequences = > Knowledge = > Capability
Multiply the opportunities for information delivery	The woman is alone or accompanied when she receives the information	Alone - By post (at the same time as the mobile unit invitation) - Reading a poster in a waiting room/town hall/shop - Visiting the Facebook page or internet site Accompanied - Flyers handed out during a collective action, with additional explanations	12: Antecedents = > Environmental context and resources = > Opportunity
Simplifying screening planning	The woman does not have to make an appointment	Send an appointment to the mobile unit (in addition to the usual breast screening invitation)	1: Goals and planning = > Goals and intentions = > Motivation
Monitoring screening participation		Access to screening participation data	2: Feedback and monitoring = > Behavioral regulation = > Capability
Monitoring the levers used to achieve the behavior		Questionnaire to assess the Com-B model dimensions (motivation, opportunity, capability)	5: Natural consequences = > Knowledge = > capability

*BCT*, behavior change techniques; *COM-B model*, capability, opportunity, motivation–behavior model; *TDF*, theoretical domain framework

The methodologies employed for measuring qualitative outcomes and defining intervention mechanisms were anticipated and operationalized through questionnaires.

## Results

### Intervention theory

The proposed causal model (Fig. 1) begins with the determinants of non-participation in breast cancer screening divided into three multi-level categories for a multi-level approach: individual determinants (age, socioeconomic level, psychosocial factors, and level of education), meso-social determinants (social/community networks), and environmental determinants (rural setting and access to healthcare professionals). Using a multidisciplinary approach, a literature review allowed for the selection and linking of two psychosocial models of behavioral change: the COM-B Model (Michie et al., 2011) and the theory of planned behavior (Ajzen, 2011). This allows for the creation of targeted interventions for capability (C), opportunity (O), and motivation (M), which lead to effective change. This model is linked to the theory of planned behavior, whose dimensions of knowledge, attitude, and intention make it possible to integrate subjective and objective reasons for behavior.

Tables 1, 2, and 3 present the key functions listed at the individual, community, and macro levels, the contextual factors to be taken into account when proposing interventions, and the concrete actions proposed for implementation. In the last column of the tables, the key function has been categorized as a BCT and linked via the TDFs to a dimension of the COM-B model by using pre-established links (Cane et al., 2015; Michie et al., 2013).

**Table 2:** Community reinforcement actions

Key functions	Context	Implementation	BCT = > TDF = > COM-B model
Mobilization of prevention stakeholders	- Adapting to local conditions and the availability of stakeholders - Mobilizing stakeholders and associations with a prevention mission	Contacts and meetings with: screening managing structure, League Against Cancer, rural families, Agricultural Social Security, health insurance	3: Social support = > Emotions = > Motivation 3: Social support = > Social influences = > Opportunity 7: Associations = > Environmental context and resources = > Opportunity
Mobilizing healthcare professionals	- Rural areas	Contacts and meetings with: - Nurse - Physician - Pharmacist	
Municipality mobilization		Contacted by the department to which they belong - Municipal action center/town halls/local shops relayed information about the arrival of the mobile unit	
Stakeholder’s training/raising awareness of literacy	- Adapting to the skills of stakeholders - To bring added value to our tools, we can think that distributing a tool, even an adapted one, without empathy and without explanations is not the most effective…	Creation of a MOOC with - Presentation of the decision aid tool - Awareness of literacy	
Adapting actions to the context and the protocol		Overview, contacts and meetings to -Identifying stakeholders who can be mobilized - Sizing information actions to target women in intervention areas	
Monitoring collective information actions	- Where - When - Who - How	- Creation of a database to record actions taken - Creation of a questionnaire for stakeholders	

*BCT*, behavior change techniques; *COM-B Model*, capability, opportunity, motivation–behavior model; *TDF*, theoretical domain framework

**Table 3:** Reinforcement actions for access to organized breast cancer screening

Key functions	Context	Implementation	*BCT* = > *TDF* = > *COM-B model*
Encourage the dissemination of information on screening	- Informing more rural women - Adapting to the context of the action	Multiplying information media: - Flyers - Posters - Video to present the mobile unit - Facebook page	12: Antecedents = > Environmental context and resources = > Opportunity
Involvement and mobilization of regional health policy stakeholders	- Division of the territory into priority zones - Taking account of stakeholders' areas of action	Involvement of departmental services Communication on the project to the population Technical service for mobile unit maintenance	
Facilitating access to services	Distance from the radiology center is associated with lower participation in screening	-The Mammobile is offered to women who live at least 15 min from an approved radiology center -Proposition of the same screening	

*BCT*, behavior change techniques; *COM-B model*, capability, opportunity, motivation–behavior model; *TDF*, theoretical domain framework

### Key functions

The first column of each table describes the 15 key functions at the interface between the determinants and levers described in the causal model.

Six were for individual reinforcement actions. These functions focused on providing information to women. They aimed to increase their capacity to participate in screening by improving their knowledge of the screening process and its benefits and risks.

Six were for community reinforcement actions. These functions involved setting up and mobilizing a network of local stakeholders to provide information as closely as possible to women, with the need both to adapt to the context and to the available partners, and to standardize actions for the purposes of evaluation.

Three were for macro-reinforcement actions for access to organized breast cancer screening. The main intervention was mobile unit integration into the screening organization, which mobilizes the structures responsible for the screening organization.

### Context

The concept of context does not have a universally accepted definition and presents a particular challenge for delineation in interventional research. We identified several elements that may interact with the interventions, some of which were inherent to the study design. In the second column of each table, the main context elements are the place in which the intervention was implemented, such as rural areas, and its consequences on healthcare professionals’ accessibility. Additional considerations included the target population’s characteristics: their socioeconomic and literacy levels, and the set of stakeholders who could be involved in their area of influence.

### Stakeholder’s typology

A typology of stakeholders who could be engaged during the intervention was established and is presented in Table 4. At the macro level, departments (administrative units) use their communication services to broadly disseminate information on the mobile units. Locally, municipalities and health services disseminate information about cancer screening and the mobile unit’s schedule. Concurrently, organizations dedicated to cancer prevention conducted informational actions. Two types of actions emerged, with information delivered either individually or collectively. These actions were constrained by the imperative to minimize crossover effects between the intervention and control arms despite their geographical proximity.

**Table 4:** Stakeholder’s and actions typology

Stakeholder’s typology	Stakeholders	Actions
Departments	Communication department	Information relay via the press Mobilization of elected representatives
Municipalities	Local social action center (prevention, lists of vulnerable people)	- Postings in the town hall, community halls, and local shops - Targeted actions as “baguette” bag
Health services	General practitioners Nurses Pharmacist Midwife	- Postings in practitioners office - Individual or group discussions on screening
Association with a prevention mission	Screening management structure Agricultural Social Security Rural families League Against Cancer	- Individual information (flyers) - Collective information

### Interventions

**Fig. 2:**
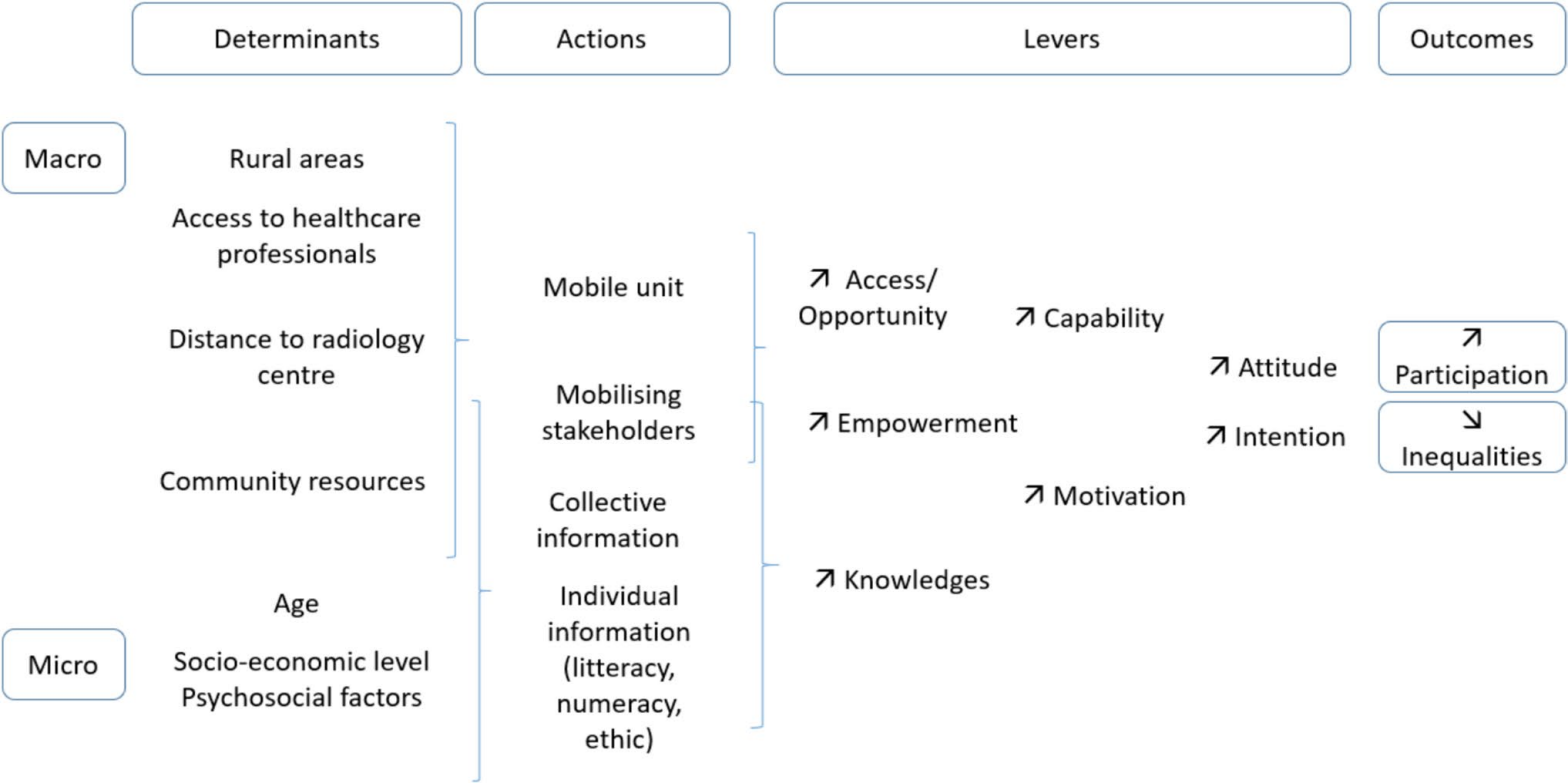
Mammobile project intervention theory

The final intervention theory for the Mammobile project is shown in Fig. 2.

This was a randomized controlled cluster trial to obtain the highest level of proof for our results. Geographic areas designated as Ilots Regroupés pour l’Information Statistique (IRIS = smallest geographical unit for which census data are available) and located more than 15 min from an approved radiology center were identified. Interventions concerned remote areas according to the principle of proportionate universalism. The mobile unit will only be offered to women who live far from a radiology center in order to reduce inequalities in access and achieve equity. These areas were grouped into clusters by combining adjacent IRIS’s until a zone with an adequate population of eligible women was formed, corresponding to around 240 eligible women, thus facilitating the 2-day presence of the mobile unit and supporting screening accessibility. A number of 30 mammograms per day was expected, determined thanks to the mobile unit of the Orne department. Subsequently, these zones were randomized into either the intervention or control arms. In total, 178 zones per arm were created.

In the control arm, eligible women received a standard invitation to undergo breast cancer screening. Accompanying the invitation, a list of approved radiology centers was included, requiring women to initiate contact to schedule an appointment. Additionally, an information brochure published by the National Cancer Institute was included.

The Mammobile project ran from March 2022 to October 2023 and was carried out on 142 intervention areas corresponding to 38,382 women invited and 178 control areas corresponding to 49,067 women invited.

In the intervention arm, in addition to the standard invitation, the interventions proposed focused on two principal objectives: improving screening accessibility and improving women’s information through a variety of comprehensive actions. From an organizational perspective, the mobile unit appointments sent to rural women represented complementary screening modalities. This outreach approach may enhance accessibility and opportunity, as well as act on the capability to screen participation.

This invitation also contained a decision-aid tool to enhance women’s knowledge and empowerment in order to motivate them. It was conceived by integrating the literacy principle with ethical imperatives to provide comprehensive data detailing both the advantages and potential risks associated with breast cancer screening.

This individual intervention was reinforced prior to the mobile unit’s visit by information actions for women, always to enhance their knowledge and empowerment about breast cancer screening and the mobile unit, and to act on motivation. This information was disseminated through a network of stakeholders specific to each area. This network also enhanced community empowerment. Thus, the departments announced the arrival of the mobile unit via their communication networks and mobilized the municipalities in the intervention zones. Details regarding the forthcoming arrival of the mobile unit were disseminated via an array of media, including posters, leaflets, and Facebook posts, which were further propagated by healthcare professionals and local social action centers. Collective information actions were organized in municipalities by some prevention organizations: screening coordination structures, health insurance, agricultural social security, and the League against Cancer. Furthermore, a Massive Open Online Course was provided to these actors to deepen their understanding of literacy issues, behavioral motivators, and ethical considerations.

A website was also developed to centralize information on the research project, mobile unit schedule, and typical breast cancer screening information. Furthermore, a video was produced and made accessible on the website, offering a visual representation of the screening process within the mobile unit.

### Evaluation

As evidence-based and data-driven action is a key element in tackling inequalities and guiding policy decisions, the intervention theory led to the development of three questionnaires:

(i) a questionnaire to assess whether our intervention increased women’s knowledge of the intervention arm and their ability to make informed choices (Hersch et al., 2015; Marteau et al., 2001) without decisional conflicts (Légaré et al., 2010)
(ii) a questionnaire to assess women’s satisfaction with screening in the mobile unit; and
(iii) a questionnaire based on the COM-B model dimensions adapted from the one proposed by Michie for the pre-intervention phase. This questionnaire contained six dimensions of the model: psychological capability, physical capability, physical opportunity, social opportunity, reflective motivation, and automatic motivation. However, it has to be validated. It has never been used for breast cancer screening or to identify the effects of interventions and the levers mobilized by women in the context of an interventional research. This questionnaire was disseminated to women in the intervention arm during the intervention, regardless of whether they came to the Mammobile.

## Discussion

To the best of our knowledge, this is the first intervention theory developed for a mobile mammography unit as part of organized breast cancer screening. This is the first use of the COM-B model for evaluation purposes. A causal model with potential mechanisms and components was proposed, and levers for enhancing women’s accessibility to screening were identified. Information dissemination was also investigated. A typology of stakeholders and their actions was delineated, with the need to develop new tools to support these actions. Finally, this theory represents a reflective basis for evaluating the project and mobile unit transferability in other contexts.

Nevertheless, the categorization of the key functions in BCTs remains subjective, and the association established with the TDFs and the dimensions of the COM-B model need to be confirmed, particularly using questionnaires. Although this approach is essential for defining initial interventions, it remains theoretical. When applied in real life, adaptation is necessary for the interventions throughout the project.

### Implications

Our theoretical model not only served as a framework for the implementation of the intervention but also established the basis for the comprehensive and complex evaluation of the project. This assessment may provide evidence-based results regarding the effectiveness of the mobile unit and its applicability and transferability to other contexts. However, between the interventions initially described (Guillaume et al., 2022) and those actually implemented, adjustments, and cancellations were necessary. These are the consequences of adapting our intervention to the context and foreseeable hazards. Women’s engagement with the decision-aid tool and information actions was markedly low, with many consequences for the process evaluation. Finally, a few actions were carried out, approximately ten in total, with only one or two women coming forward. However, these effects have not been evaluated.

Information remains a key factor in participation in breast cancer screening programs. Semi-structured interviews were conducted during the intervention to elucidate women’s information preferences regarding breast cancer screening and understand why women did not attend the information meetings and did not use the decision-making tool specially designed for the project. Despite the widespread dissemination of questionnaires across the two protocol arms in both printed and digital formats, the return rates were not as high as expected (258 for the informed choice questionnaire and 204 for the satisfaction questionnaire). The questionnaire assessing the dimensions of the COM-B model yielded 137 responses, marked by a substantial incidence of incomplete data. The dual objectives of this questionnaire were validation and analysis of participant responses; however, the sample size was inadequate for these purposes. Nonetheless, we were optimistic about disseminating this questionnaire through a network of women who volunteered for research initiatives. The evaluation of the intervention and its underlying mechanisms, defined a priori, remains a complex undertaking within an interventional framework that resists standardization throughout its duration.

The project is now in its evaluation phase and, in line with the intervention theory approach, the hypotheses used and the choices adopted for this intervention will be compared with real interventions to draw conclusions in terms of public health for the subsequent transferability of the mobile unit device in other contexts.

### Implications for policy and practice

What are the innovations in this policy or program?

- The construction of a conceptual model for increasing breast cancer screening participation using a mobile mammography unit combined with targeted information.

- The use of the COM-B model remains original in understanding and acting on women’s adherence to organized breast cancer screening.

What are the burning research questions for this innovation?

- Are information and empowerment still key factors in women’s adherence to organized breast cancer screening?

- Can the model’s causal mechanisms be validated?

## Data Availability

Not applicable.

## Abbreviations

BCT: Behavior change techniques
COM-B: Model: capability, opportunity, motivation-behavior model
FIC model: Functions key, implementation, context model
IRIS: Ilots Regroupés pour l’Information Statistique
TDF: Theoretical domain framework
